# BRD4 inhibitor reduces exhaustion and blocks terminal differentiation in CAR-T cells by modulating BATF and EGR1

**DOI:** 10.1186/s40364-024-00667-w

**Published:** 2024-10-15

**Authors:** Songnan Sui, Mengjun Zhong, Shuxin Zhong, Xueting Peng, Lipeng Mao, Cunte Chen, Chengwu Zeng, Oscar Junhong Luo, Yangqiu Li

**Affiliations:** 1 Department of Hematology, First Affiliated Hospital, Jinan University, Guangzhou, China; 2 Key Laboratory for Regenerative Medicine of Ministry of Education, Institute of Hematology, School of Medicine, Jinan University, Guangzhou, China; 3 Department of Systems Biomedical Sciences, School of Medicine, Jinan University, Guangzhou, China; 4 Department of Hematology, Guangzhou First People’s Hospital, Institute of Blood Transfusion and Hematology, Guangzhou Medical University, Guangzhou, China; 5 Central People’s Hospital of Zhanjiang, Zhanjiang, China; 6 Zhanjiang Key Laboratory of Leukemia Pathogenesis and Targeted Therapy Research, Zhanjiang, China

**Keywords:** Chimeric antigen receptor T cell exhaustion, BRD4 inhibitor, Single-cell RNA sequencing, Transcriptional factors

## Abstract

**Background:**

Exhaustion is a key factor that influences the efficacy of chimeric antigen receptor T (CAR-T) cells. Our previous study demonstrated that a bromodomain protein 4 (BRD4) inhibitor can revise the phenotype and function of exhausted T cells from leukemia patients. This study aims to elucidate the mechanism by which a BRD4 inhibitor reduces CAR-T cell exhaustion using single-cell RNA sequencing (scRNA-Seq).

**Methods:**

Exhausted CD123-specific CAR-T cells were prepared by co-culture with CD123 antigen-positive MV411 cells. After elimination of MV411 cells and upregulation of inhibitory receptors on the surface, exhausted CAR-T cells were treated with a BRD4 inhibitor (JQ1) for 72 h. The CAR-T cells were subsequently isolated, and scRNA-Seq was conducted to characterize phenotypic and functional changes in JQ1-treated cells.

**Results:**

Both the proportion of exhausted CD8^+^ CAR-T cells and the exhausted score of CAR-T cells decreased in JQ1-treated compared with control-treated cells. Moreover, JQ1 treatment led to a higher proportion of naïve, memory, and progenitor exhausted CD8^+^ CAR-T cells as opposed to terminal exhausted CD8^+^ CAR-T cells accompanied by enhanced proliferation, differentiation, and activation capacities. Additionally, with JQ1 treatment, BATF activity and expression in naïve, memory, and progenitor exhausted CD8^+^ CAR-T cells decreased, whereas EGR1 activity and expression increased. Interestingly, AML patients with higher EGR1 and EGR1 target gene ssGSEA scores, coupled with lower BATF and BATF target gene ssGSEA scores, had the best prognosis.

**Conclusions:**

Our study reveals that a BRD4 inhibitor can reduce CAR-T cell exhaustion and block exhausted T cell terminal differentiation by downregulating BATF activity and expression together with upregulating EGR1 activity and expression, presenting an approach for improving the effectiveness of CAR-T cell therapy.

**Supplementary Information:**

The online version contains supplementary material available at 10.1186/s40364-024-00667-w.

## Background

Acute myeloid leukemia (AML) is the most common type of leukemia in adults [1]. Although most AML patients achieve complete remission after standard chemotherapy, refractory and relapsed disease remain an issue [2]. Patients with refractory or relapsed disease have poor prognosis with an overall 5-year survival rate of less than 30% [3]. Over the past decade, immunotherapy has been widely applied in clinical cancer treatment. Chimeric antigen receptor T (CAR-T) cell therapy has been identified as a highly promising immunotherapeutic strategy, demonstrating remarkable success in the treatment of hematological malignancies, particularly B cell malignancies [4, 5]. This type of immunotherapy can improve prognoses of patients suffering from AML, including those for whom the current standard therapeutic approaches have proven ineffective [6]. However, CAR-T cell therapy faces some challenges. CAR-T cell exhaustion is considered a major limiting factor for the efficacy of CAR T-cell therapy [7]. Mitigating exhaustion to maintain the effector function and persistence of CAR-T cells, thereby achieving durable clinical efficacy, remains a core challenge.

T cells play a protective role in the body, tasked with the elimination of recognized antigens [8]. However, under certain circumstances, the function of T cells may be suppressed by the effects of T cell exhaustion [9]. T cell exhaustion may arise from factors such as persistent exposure of T cells to antigens, the microenvironment surrounding the T cells, and immunosuppressive cells [10]. The characteristics of exhausted T cells include increased expression of inhibitory receptors, such as programmed cell death 1 (PD-1), T cell immunoglobulin mucin-domain-containing-3 (TIM-3), lymphocyte activation gene-3 (LAG-3), and cytotoxic T-lymphocyte associated protein 4 (CTLA4) as well as metabolic dysregulation, functional impairment, and alterations in key transcription factor (TF) genes [11]. Similarly, CAR-T cell exhaustion is also believed to be driven by sustained tumor antigen stimulation and an immunosuppressive tumor microenvironment, leading to transcriptional and epigenetic changes [12]. Furthermore, CAR-T cell exhaustion can also impact their function and is even associated with prognosis [13]. For instance, in patients with B-cell lymphomas that failed to respond to treatment, CD19 CAR-T cells tended to exhibit the exhausted phenotype [14]. Single-cell RNA sequencing (scRNA-Seq) analysis of CD19 CAR-T cells used in therapy for patients with B cell lymphomas has also revealed that patients with partial or no remission exhibited a higher prevalence of exhaustion-related transcriptional profiles compared to those who achieved complete remission [15]. Studies have also shown that PD-1/PD- ligand 1 (PD-L1) blockade can improve the efficacy of CAR-T cell therapy [16]. These findings underscore the critical role of CAR-T cell exhaustion as a major factor limiting the therapeutic potential of this approach. Overcoming CAR-T cell exhaustion is a crucial challenge that must be addressed to improve the efficacy of CAR-T cell therapy for hematological malignancies.

T cell exhaustion is a multistage, progressive process that exhibits a remarkable degree of heterogeneity [17]. Furthermore, T cells in different stages of exhaustion display distinct epigenetic and transcriptional characteristics [18]. An early study has reported that T cell exhaustion involves two distinct chromatin states: a reversible dysfunctional state from which T cells can be rejuvenated and an irreversible dysfunctional state in which T cells are recalcitrant to therapeutic reprogramming [19]. Recent studies have indicated that exhausted T cells can be divided into several distinct stages: progenitor, intermediate, terminal, and proliferating exhausted [20, 21]. Progenitor exhausted T cells with higher expression levels of signature genes associated with naïve and memory T cells are easy to be reprogrammed and can continue to proliferate even after PD-1/PD-L1 blockade [21]. In contrast, terminal exhausted T cells, marked by higher expression levels of inhibitory receptors, such as *PD-1* and *TIM-3*, are unable to be reprogrammed and lose proliferative capacity after PD-1/PD-L1 blockade [22]. The heterogeneity observed in exhausted T cells also extends to exhausted CAR-T cells. Exhausted CAR-T cells can also be subdivided into distinct subpopulations: intermediate exhausted CAR-T and terminal exhausted CAR-T cells. These subpopulations have distinct epigenetic landscapes, as determined by single-cell sequencing of transposase-accessible chromatin [23].

The BET protein family of TFs, including bromodomain protein (BRD) 2, BRD3, BRD4, and bromodomain testis-specific protein (BRDT), are important for maintaining homeostasis [24]. Within this protein family, BRD4 is the most widely studied member. An increasing number of studies have revealed that dysfunctional BET proteins, particularly BRD4, will lead to gene mutations and abnormal cell proliferation, resulting in malignant cell proliferation and carcinogenesis [25]. In recent years, anti-tumor drugs targeting BET proteins have shown remarkable effects in preclinical studies and clinical trials [26]. JQ1, a first-generation synthetic BET inhibitor, displayed significant antitumor activity in a mouse model and the possibility of clinical transformation in early clinical trials against hematologic malignancies [27]. In addition to direct inhibitory effects in tumor cells, recent research has found that BET inhibitors also inhibit the progression of malignancies by improving the anti-tumor immune response. JQ1 can maintain the properties of central memory T (Tcm) cells and enhance the functional persistence of CD8^+^ T cells by directly inhibiting basic leucine zipper ATF-like transcription factor (BATF), a regulatory factor related to phenotypic differentiation of effector memory cells in CD8^+^ T cells [28]. Our previous study has demonstrated that a BET inhibitor could suppress the expression levels of inhibitory receptors on CAR-T cells. Furthermore, CAR-T cells treated with JQ1 exhibited reduced exhaustion and enhanced activity [29]. However, the mechanism by which JQ1 enhances the anti-tumor efficacy of CAR-T cells is not fully understood.

Therefore, in this study, we co-cultured CD123 CAR-T cells with a CD123-expressing AML cell line until the exhausted phenotypes emerged on the surface of the CAR-T cells, and these cells were then treated with JQ1. Subsequently, scRNA-Seq and single-cell T cell receptor sequencing (scTCR-Seq) were performed to elucidate the mechanisms by which JQ1 reduces CAR-T cell exhaustion.

## Methods

### Construction of a CAR-T cell exhaustion model and experimental diagram

**Fig. 1:**
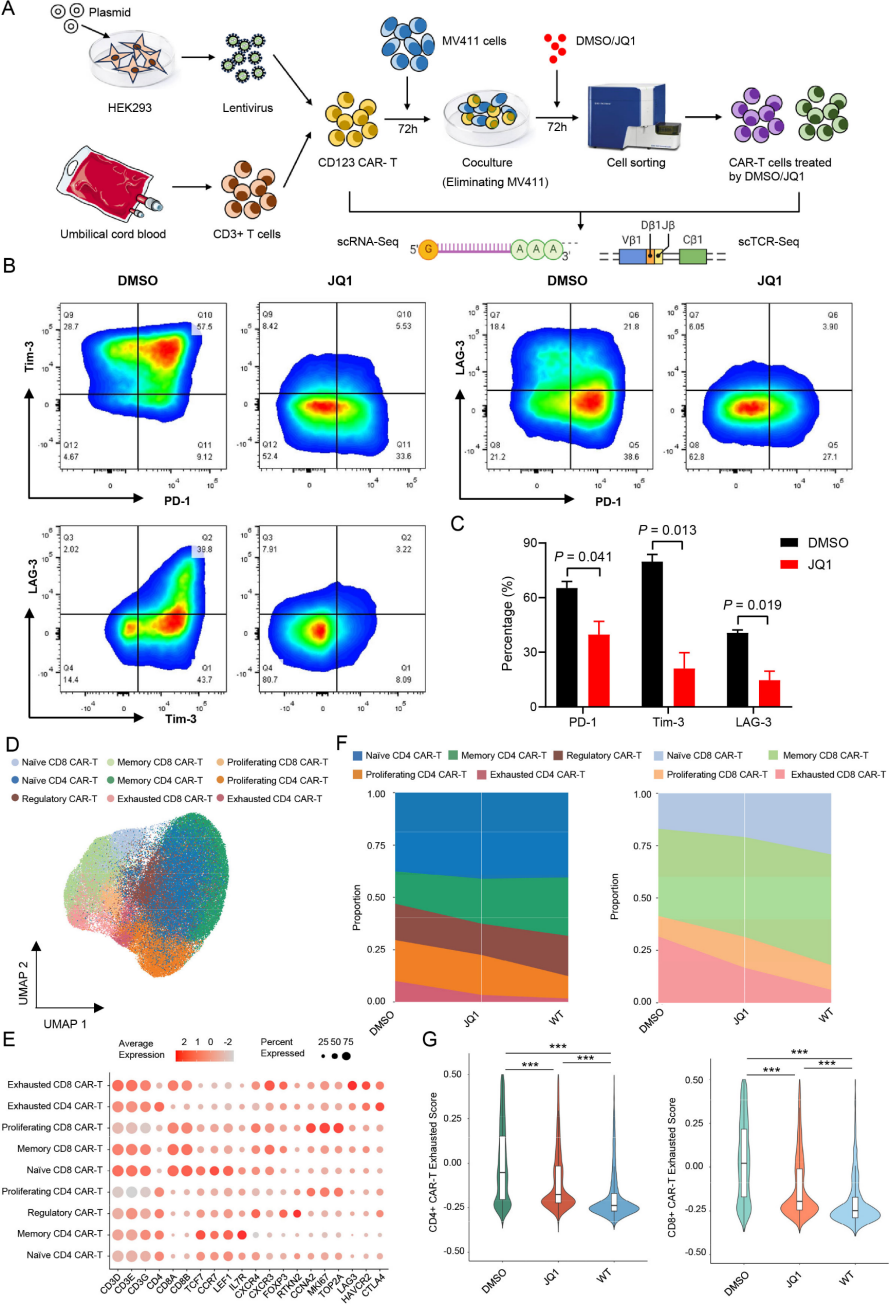
JQ1 reduces CAR-T cell exhaustion (**A**) Study design schematics (**B**-**C**) After treatment with JQ1 for 72 h, the percentages of PD-1, Tim-3, and LAG3 on CAR-T cells were determined by flow cytometry (*n* = 3) (**D**) UMAP (Uniform Manifold Approximation and Projection) plot of CAR-T cell scRNA-seq datasets color coded by nine distinct cell types (**E**) Dot plot for marker genes of each CAR-T cell type. Color gradient represents the average scaled expression of marker genes in each cell type, and dot size depicts the percentage of cells expressing the marker genes in each cell type (**F**) Proportion of subpopulations within the CD4^+^ (left) and CD8^+^ (right) CAR-T cell populations (**G**) Violin plots of the exhausted scores for CD4^+^ (left) and CD8^+^ (right) CAR-T cells

In our previous study, we successfully constructed an in vitro model for CAR-T cell exhaustion. Briefly, CD123 CAR-T cells and MV411 cells with high CD123 expression were co-cultured for 72 h according to an effect-target ratio of 1:3 − 1:4, and the co-cultured cells were collected and analyzed by flow cytometry to detect the expression of CD123, PD-1, Tim-3, and LAG3. After confirming that the MV411 cells were cleared and the CAR-T cells were exhausted, the exhausted T cells were divided into two groups and treated with DMSO as control and 0.2 µM JQ1 for 72 h. After treatment, the exhausted CAR-T cells and non-exhausted CAR-T cells were sorted by flow cytometry, and GFP + CAR-T cells were selected and sent for scRNA-Seq and scTCR-Seq (Fig. 1A).

### scRNA-Seq and scTCR-Seq library construction

scRNA-seq and scTCR-Seq libraries were generated using 10X Genomics Chromium Single Cell 5’ in addition to the V(D)J enrichment reagent kit per the manufacturer’s protocols. Sequencing was performed utilizing the Illumina NovaSeq 6000 platform.

### scRNA-Seq data quality control and processing

scRNA-Seq raw data were mapped to the human GRCh38 reference genome using the CellRanger v6.1.2 pipeline, resulting in cell-by-gene expression matrices. The expression matrices were then processed using Seurat v4.3.0 for quality control and filtering. Genes detected in fewer than three cells were removed, and cells were selected according to predetermined parameters to preserve high-quality data for subsequent analysis. The selection criteria for the retained cells included a minimum of 800 and a maximum of 6,000 detected genes as well as a mitochondrial gene proportion below 10%.

### scRNA-Seq data clustering and annotation

The filtered scRNA-Seq dataset was processed using the Seurat v4.3.0 package for clustering and visualization. The NormalizeData function was applied to normalize the gene expression data, and the top 2,000 variable genes were selected using the FindVariableFeatures function. Next, the ScaleData function was used to scale the gene expression data, incorporating a regression to remove cell cycle-associated variations. Dimensionality reduction was performed using the RunPCA function, and further non-linear dimensionality reduction was performed with the RunUMAP function. Clustering of the cells was conducted by the FindClusters function. The cell clusters were annotated based on highly expressed signature genes in each cluster. Batch effects were mitigated, and samples were merged through application of the Harmony v0.1.1 package.

### Exhausted and proliferative scores

Exhausted and proliferative scores were generated based on scRNA-Seq data using the AddModuleScore function in the Seurat v4.3.0 package. Exhausted and proliferative scores were calculated according to gene sets related to exhaustion and proliferation, respectively.

### Gene set functional enrichment analysis

The ClusterProfiler v4.2.2 package was employed to perform functional enrichment analysis of marker genes [30]. Gene Ontology (GO) and Kyoto Encyclopedia of Genes and Genomes (KEGG) enrichment analyses were accomplished by the enricher function based on the GO and KEGG gene sets in the Molecular Signatures Database (MsigDB) database. Gene set enrichment analysis (GSEA) was conducted using the GSEA function with the C2, C5, and C7 gene sets sourced from the MSigDB database.

### Gene regulatory network analysis

Pyscenic v0.12.1 was used to analyze the gene regulatory networks of the different CAR-T cell subgroups [31]. The gene regulatory network (GRN) construction workflow involved several key steps. First, the grn function was employed to build the initial regulatory network, which linked TF genes to their putative target genes based on co-expression analysis. To refine the initial network and improve the reliability of the TF-target gene associations, the ctx function was applied. This function considered both the motif-TF relationships and the rankings of the motif regulatory potential of the genes, effectively trimming the network to retain only the most robust and biologically relevant connections. Finally, the aucell function was used to evaluate the activity of each regulon (a TF and its target genes) in a single cell. Furthermore, downstream analyses, such as extraction of regulatory network data and selection of TF with differential activity among different groups, were performed using the SCENIC v1.2.4 package.

### Single-sample gene set enrichment analysis

The single-sample gene set enrichment analysis (ssGSEA) algorithm from the GSVA v1.42.0 package was used to calculate the enrichment scores for various gene sets within each individual AML patient sample from The Cancer Genome Atlas (TCGA) database [32]. The ssGSEA CD8_Naïve_EGR1 score, ssGSEA CD8_Memory_EGR1 score, and ssGSEA progenitor CD8_Ex_EGR1 score represented the ssGSEA scores calculated based on *EGR1* and its top 30 differentially expressed target genes in CD8_Naïve, CD8_Memory, and progenitor CD8_Ex CAR-T cells, respectively, in the AML patient dataset. Similarly, the ssGSEA CD8_Naïve_BATF score, ssGSEA CD8_Memory_BATF score, and ssGSEA progenitor CD8_Ex_BATF score were calculated by the ssGSEA algorithm according to *BATF* and its top 30 differentially expressed target genes in CD8_Naïve, CD8_Memory, and progenitor CD8_Ex CAR-T cells, respectively.

### Prognostic analysis

X-tile software v3.6.1 was utilized to calculate the prognostic cut-off thresholds for ssGSEA scores. Kaplan-Meier survival curves were plotted to visualize the overall survival (OS) of AML patients using the survival v3.5-5 and survminer 0.4.9 packages. SPSS was employed to establish both univariate and multivariate cox regression models for the survival analysis. Statistical significance was defined as *P* value less than 0.05.

### scTCR-Seq data quality control and processing

scTCR-seq raw data were processed using the VDJ pipeline of Cell Ranger, which was obtained from the 10x Genomics website. Human genome GRCh38 was used as reference for analysis. Then, the matrix containing the obtained TCR sequence information for each cell was filtered based on the principle of only retaining αβ paired TCR clonotypes. Subsequent analysis of the TCR information was performed using the scRepertoire v1.8.0 package [33]. The combine TCR function was employed to merge the data from all samples. The quant Contig function was used to quantify the relative percentage of unique TCR clonotypes. The total numbers of clonotypes across the different samples were identified by the abundance Contig function. To assess the TCR clonotypic diversity, the clonal Diversity function was utilized to calculate the Chao index and Inverse Simpson index.

## Results

### JQ1 decreases CAR-T cell exhaustion

After co-culture of CD123 CAR-T cells with MV411 cells, expression of the immunosuppressive receptors PD-1, Tim-3, and LAG-3 on the CAR-T cells was significantly upregulated. However, expression of these proteins was significantly decreased by administration of 0.2 µM JQ1 (Fig. 1B, C).

After integration, dimensionality reduction, and clustering analysis of scRNA-Seq data, CAR-T cells were divided into nine distinct cell clusters. These clusters were then annotated based on marker gene expression, including exhausted CD4^+^ and CD8^+^ (CD4_Ex and CD8_Ex) CAR-T cells marked with high expression of genes (*LAG-3*,* HAVCR2*, and *CTLA-4*) related to exhaustion (Fig. 1D, E and Fig. S1A). Importantly, the proportions of naïve CD4^+^ (CD4_Naïve), memory CD4^+^ (CD4_Memory), naïve CD8^+^ (CD8_Naïve) and memory CD8^+^ (CD8_Memory) CAR-T cells were higher, while the proportions of CD4_Ex and CD8_Ex CAR-T cells were lower in JQ1-treated cells compared to DMSO-treated (control) cells (Fig. 1F). Furthermore, exhausted scores for CD4^+^ and CD8^+^ CAR-T cells were notably lower in the JQ1 group compared to the control group (Fig. 1G). Additionally, the expression of exhaustion marker genes was also elevated in JQ1-treated CAR-T cells (Fig. S1B). Moreover, CD4^+^ and CD8^+^ CAR-T cells in the JQ1 group exhibited a stronger proliferative capacity compared to the control group (Fig. S1C). These findings suggested that a BRD4 inhibitor can effectively mitigate CAR-T cell exhaustion.

### JQ1 enhances the function of naïve and memory CD8^+^ CAR-T cells

**Fig. 2:**
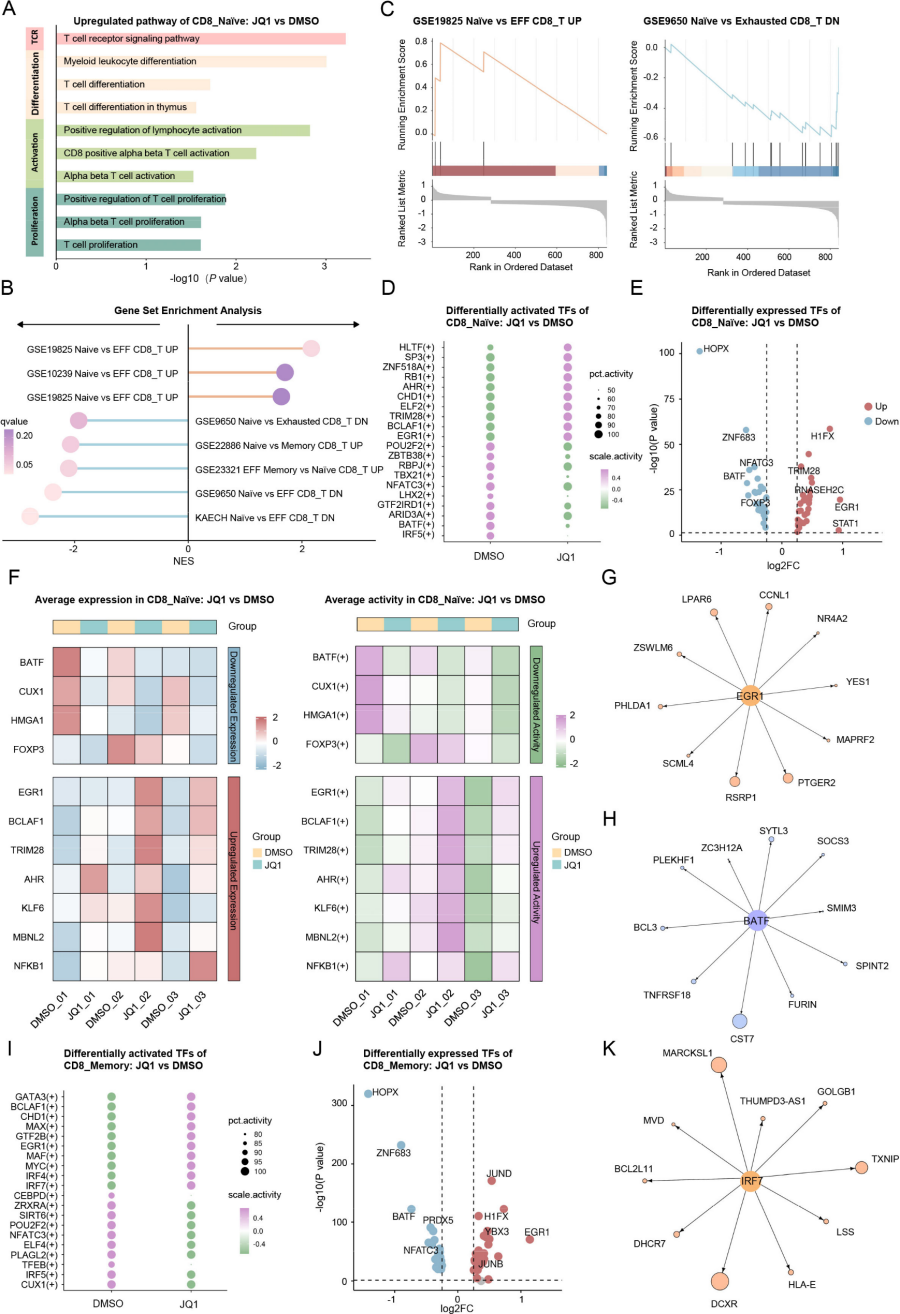
JQ1 reprograms the transcriptional profiles of CD8^**+**^**naïve and memory CAR-T cells** (**A**) KEGG and GO enrichment plot for genes upregulated in CD8^+^ naïve CAR-T cells in the JQ1 treated group (**B**) GSEA (Gene Set Enrichment Analysis) of genes upregulated in CD8^+^ naïve CAR-T cells in the JQ1 compared to DMSO group (**C**) GSEA plot showing the expression enrichment of genes related to the GSE19825 Naïve vs. EFF CD8_T UP (left) and GSE9650 Naïve vs. Exhausted CD8_T DN (right) pathways in CD8^+^ naïve CAR-T cells from the JQ1 group (**D**) Dot plot of the top 10 differentially active transcription factors (TFs) in CD8^+^ naïve CAR-T cells treated with JQ1 or DMSO (**E**) Volcano plot of TF genes differentially expressed in CD8^+^ naïve CAR-T cells treated with JQ1 vs. DMSO (**F**) Heatmap displaying the expression levels (left) and activities (right) of TFs that are consistently upregulated or downregulated in both expression and activity in CD8^+^ naïve CAR-T cells with JQ1 treatment (**G**) Network diagram of the EGR1 regulon (EGR1 and its top 10 target genes) in CD8^+^ naïve CAR-T cells. The edge length from EGR1 to a target gene is proportional to the regulation weight by EGR1. The target gene node size represents the difference in the level of expression between JQ1-treated and DMSO-treated cells (**H**) Network diagram of the BATF regulon (BATF and its top 10 target genes) in CD8^+^ naïve CAR-T cells. The edge length from BATF to a target gene is proportional to the regulation weight by BATF. The target gene node size represents the difference in the level of expression between JQ1-treated and DMSO-treated cells (**I**) Dot plot of the top 10 differentially actived TFs in CD8^+^ memory CAR-T cells treated with JQ1 vs. DMSO (**J**) Volcano plot of differentially expressed TF genes in CD8^+^ memory CAR-T cells treated with JQ1 vs. DMSO (**K**) Network diagram of the IRF7 regulon (IRF7 and its top 10 target genes) in CD8^+^ memory CAR-T cells. The edge length from IRF7 to a target gene is proportional to the regulation weight by IRF7. The node size of the target genes represents difference in expression level between JQ1-treated and DMSO-treated cells

Given the higher proportions of CD8_Naïve and CD8_Memory CAR-T cells in the JQ1 treatment group, we conducted a more comprehensive investigation into the effects of JQ1 on the phenotype and function of the CAR-T cell subpopulations. First, GO and KEGG enrichment analysis was performed based on genes upregulated in CD8_Naïve CAR-T cells in the JQ1 group compared to the DMSO control group. The results revealed a significant enrichment in pathways related to T cell proliferation, activation, and differentiation and T cell receptor (TCR) signaling (Fig. 2A). To further elucidate the transcriptional profiles of JQ1-treated CD8_Naïve CAR-T cells, we conducted GSEA. The results demonstrated that JQ1-treated CD8_Naïve CAR-T cells had higher expression of genes linked to the naïve T cell phenotype and lower expression of genes associated with T cell exhaustion (Fig. 2B, C). To gain deeper insight into the underlying mechanisms by which JQ1 can induce transcriptional landscape changes in CD8_Naïve CAR-T cells, we further performed transcriptional regulatory network analysis. This analysis revealed distinct patterns of TF activity between DMSO-treated and JQ1-treated CD8_Naïve CAR-T cells (Fig. 2D). Specifically, TFs, such as early growth response 1 (EGR1) and BCL2 associated transcription factor 1 (BCLAF1) exhibited elevated activity in the JQ1 group, while POU class homeobox 2 (POU2F2) and zinc-finger and BTB domain containing 38 (ZBTB38) demonstrated higher activation in the control group. Furthermore, the expression of the TF genes differed between the two conditions (Fig. 2E). For example, the expression of *EGR1* and signal transducer and activator of transcription 1 (STAT1) increased in the JQ1 group compared to the control group. Conversely, the expression of TF genes, such as homeodomain-only protein homeobox (HOPX) and zinc finger protein 683 (ZNF683), was higher in the control group. Building on these findings, we conducted further screening to identify TFs that exhibited concordant changes in both expression and activity level between DMSO-treated and JQ1-treated CD8_Naïve CAR-T cells. This analysis revealed that in CD8_Naïve CAR-T cells from the JQ1 group, TFs such as *BATF* and CUT-like homeobox 1 (CUX1) displayed reduced expression and decreased activity levels (Fig. 2F). Conversely, TFs such as *EGR1* and *BCLAF1* demonstrated elevated expression coupled with increased activity with JQ1 treatment (Fig. 2F). GSEA analysis of the target genes regulated by *BATF* and *EGR1* revealed that genes associated with T cell proliferation, activation, and differentiation were upregulated, while genes related to apoptosis and exhaustion were downregulated (Fig. 2G, H and Fig. S2A). These results demonstrated that JQ1 may enhance the function of CD8_Naïve CAR-T cells by modulating TFs that govern key cellular processes.

Similar trends were also observed in CD8_Memory CAR-T cells between the JQ1 treatment and control groups. First, genes upregulated in JQ1-treated CD8_Memory CAR-T cells were significantly enriched for pathways associated with T cell proliferation, activation, differentiation, and TCR signaling (Fig. S2B). Further characterization by GSEA revealed that CD8_Memory CAR-T cells from the JQ1 group had an enhanced transcriptional profile with upregulation of gene signatures corresponding to naïve and memory T cell phenotypes concurrent with downregulation of genes related to T cell exhaustion (Fig. S2C, D). These results indicated that JQ1 not only promoted a more proliferative and activated state in CD8_Memory CAR-T cells but also mitigated the expression of exhaustion-associated genes. Similarly, we examined the differentially expressed and activated TFs, and identified TFs such as *BATF*, which were consistently downregulated, and TFs such as interferon regulatory factor 7 (IRF7), which were consistently upregulated, in CD8_Memory CAR-T cells treated with JQ1 (Fig. 2I, J and Fig. S2E). Enrichment analysis of the *BATF* and *IRF7* target genes also identified that genes related to T cell proliferation, activation, and differentiation were upregulated, while genes linked to exhaustion and apoptosis were downregulated (Fig. 2K and Fig. S2F, G). These findings indicated that the JQ1-mediated effects on the transcriptional landscapes were not only limited to CD8_Naïve CAR-T cells but also extend to CD8_Memory CAR-T cells.

### Maintaining the early exhausted status of CD8+ CAR-T cells with JQ1

**Fig. 3:**
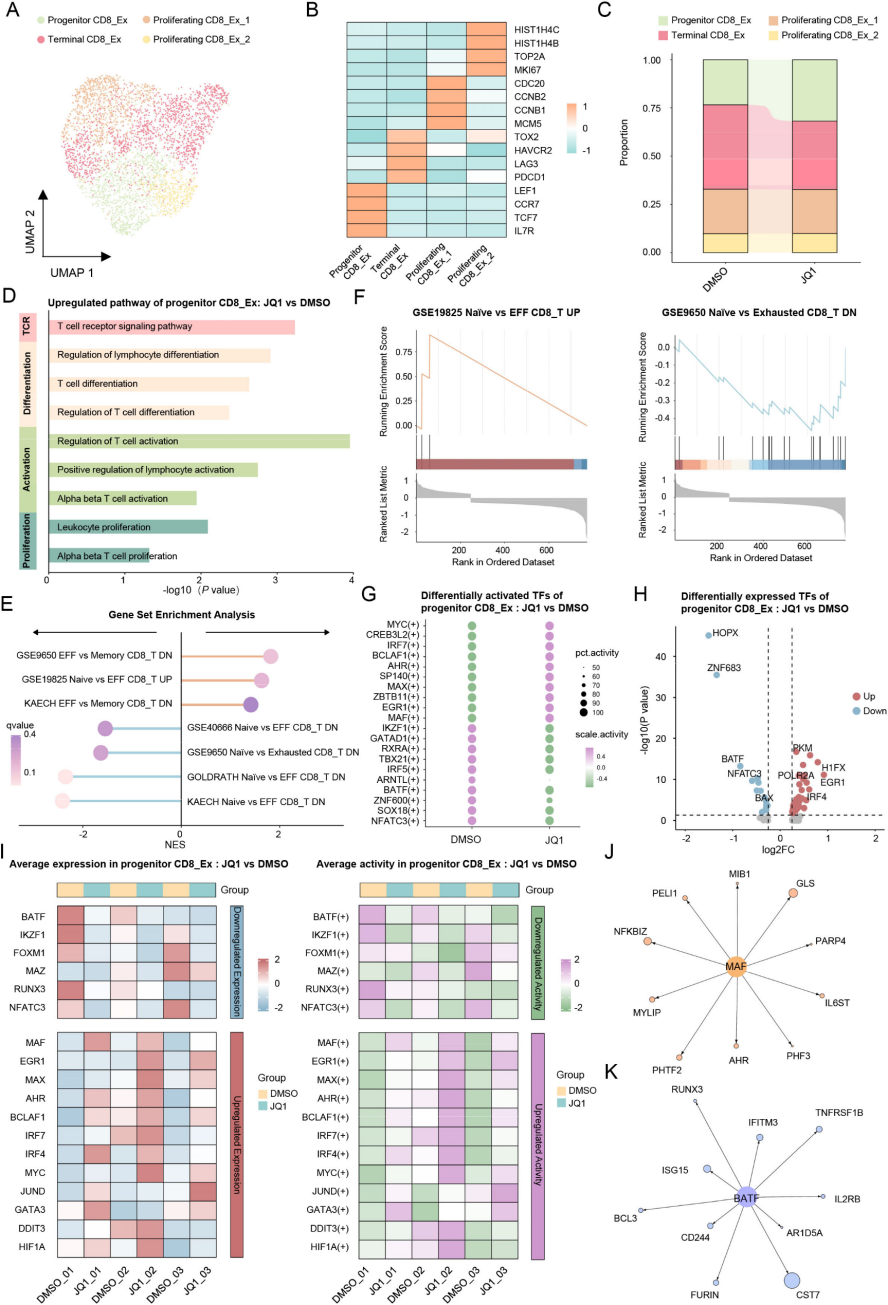
JQ1 increases the proportion and reshapes the transcriptional profile of progenitor exhausted CD8^**+**^**CAR-T cells** (**A**) UMAP plot of subpopulations of exhausted CD8^+^ (CD8_Ex) CAR-T cells color-coded by distinct cell types (**B**) Heatmap of different subpopulation signature genes in CD8_Ex CAR-T cells (**C**) Proportion of the subpopulations in CD8_Ex CAR-T cells treated with JQ1 vs. DMSO (**D**) KEGG and GO enrichment plot for genes upregulated in progenitor CD8_Ex CAR-T cells in JQ1-treated vs. DMSO-treated groups (**E**) GSEA of genes upregulated in progenitor CD8_Ex CAR-T cells in JQ1-treated vs. DMSO-treated cells (**F**) GSEA plot showing the expression enrichment of genes related to the GSE19825 Naïve vs. EFF CD8_T UP (left) and GSE9650 Naïve vs. Exhausted CD8_T DN (right) pathways in progenitor CD8_Ex CAR-T cells from the JQ1 group (**G**) Dot plot of the top 10 differentially actived TF genes between progenitor CD8_Ex CAR-T cells in the JQ1 group and progenitor CD8_Ex CAR-T cells in the DMSO group (**H**) Volcano plot of TF genes differentially expressed in progenitor CD8_Ex CAR-T cells treated with JQ1 vs. progenitor CD8_Ex CAR-T cells treated with DMSO (**I**) Heatmap displaying the expression levels (left) and activities (right) of TF genes that are consistently upregulated or downregulated in both expression and activity in progenitor CD8_Ex CAR-T cells treated with JQ1 compared to DMSO control (**J**) Network diagram of the MAF regulon (MAF and its top 10 target genes) in progenitor CD8_Ex CAR-T cells (**K**) Network diagram of the BATF regulon (BATF and its top 10 target genes) in progenitor CD8_Ex CAR-T cells

Addressing the heterogeneity within the CD8_Ex CAR-T cells is crucial, as different subgroups may exhibit varying responses to pharmacological interventions. To investigate this hypothesis, we performed dimensionality reduction and clustering of CD8_Ex CAR-T cells, which revealed three distinct subgroups, progenitor, terminal, and proliferating CD8_Ex CAR-T cells, which were identified by high expression levels of marker genes (Fig. 3A, B). Progenitor CD8_Ex CAR-T cells were characterized by their high expression of genes associated with naïve and memory T cells, such as T-cell factor 7 (TCF7) and chemokine receptor 7 (CCR7). In contrast, terminal CD8_Ex CAR-T cells demonstrated elevated levels of exhaustion-related genes including programmed cell death 1 (PDCD1), *LAG-3*, and thymocyte selection-associated high mobility group box 2 (TOX2). Proliferating CD8_Ex CAR-T cells were marked by upregulated expression of proliferation-associated genes, such as proliferation marker protein Ki-67 (MKI67), cyclin B1 (CCNB1), topoisomerase II alpha (TOP2A), and cyclin B2 (CCNB2). Notably, an increase in the proportion of progenitor CD8_Ex CAR-T cells was observed in the JQ1 group compared to the control group (Fig. 3C). Conversely, the proportion of terminal CD8_Ex CAR-T cells was decreased in the JQ1 group relative to the control group (Fig. 3C). This finding indicated that a BRD4 inhibitor may maintain the progenitor CD8_Ex CAR-T cell population and block their terminal differentiation, potentially enhancing their proliferative capacity and anti-tumor functionality.

Next, GO and KEGG enrichment analysis revealed that genes upregulated in progenitor JQ1-treated CD8_Ex CAR-T cells were associated with key processes such as T cell differentiation, activation, and proliferation and TCR signaling (Fig. 3D). GSEA results demonstrated that in JQ1-treated progenitor CD8_Ex CAR-T cells, there was an upregulation of gene signatures associated with naïve and memory T cells. At the same time, gene sets representing T cell exhaustion markers were downregulated in JQ1-treated progenitor CD8_Ex CAR-T cells (Fig. 3E, F). These results indicated that JQ1 may enhance the functional and cytotoxic potential of the progenitor CD8_Ex CAR-T cells by promoting a less-exhausted, more stem-like phenotype. TF activity analysis demonstrated that TFs, including musculoaponeurotic fibrosarcoma (MAF), *EGR1*, and zinc finger and BTB domain containing 1 (ZBTB1) had higher activity in progenitor CD8_Ex CAR-T cells in JQ1-treated cells (Fig. 3G). In contrast, TFs, including ikaros family zinc finger 1 (IKZF1), GATA zinc finger domain containing 1 (GATAD1), and retinoid X receptor alpha (RXRA), demonstrated higher activity in progenitor CD8_Ex CAR-T cells in the control group relative to the JQ1 group (Fig. 3G). Furthermore, the expression of *EGR1*, H1 histone family member X (H1FX), and pyruvate kinase M (PKM) was upregulated, while the expression of *HOPX*, *ZNF683*, and *BATF* was downregulated in progenitor CD8_Ex CAR-T cells in the JQ1 group (Fig. 3H). Notably, TFs, including *MAF* and *EGR1*, exhibited consistent upregulation of both activity and expression in the JQ1 group (Fig. 3I). In contrast, *BATF* and *IKZF1* demonstrated consistent downregulation of both activity and expression in progenitor CD8_Ex CAR-T cells in the JQ1 group (Fig. 3I). Similarly, GSEA based on *MAF* and *BATF* target genes also revealed upregulation of pathways associated with T cell differentiation, activation, and proliferation as well as downregulation of pathways related to exhaustion and apoptosis (Fig. 3J, K and Fig. S2H). These results provide mechanistic insights into how the modulation of key TFs, such as *EGR1* and *BATF*, may contribute to enhancing the functionality and expansion of progenitor CD8_Ex CAR-T cells upon JQ1 treatment.

### JQ1 may reduce CAR-T cell exhaustion by modulating the activity of BATF and EGR1

**Fig. 4:**
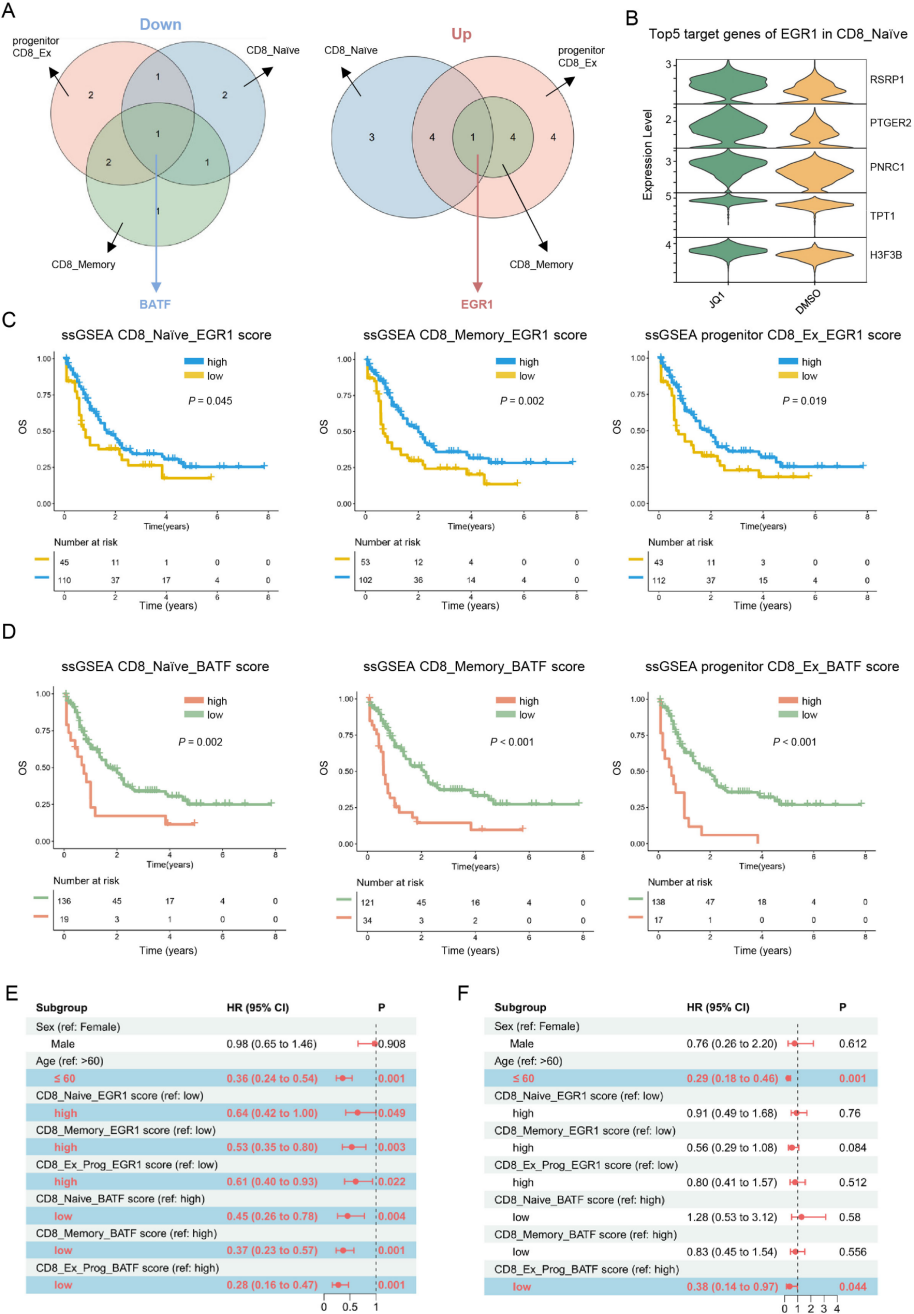
JQ1 may improve the prognosis of AML patients by influencing the activity and expression of BATF and EGR1 (**A**) Venn diagram representing the intersection of differentially expressed and activated TFs among CD8^+^ naïve, CD8^+^ memory, and progenitor CD8_Ex CAR-T cells in JQ1-treated compared to DMSO-treated cells (**B**) Violin plots showing the expression levels of the top 5 differentially expressed EGR1 target genes in CD8^+^ naïve CAR-T cells (**C**) Overall survival (OS) for the low and high ssGSEA (single-sample Gene Set Enrichment Analysis) score subgroups of AML patients from the TCGA-AML dataset (left: ssGSEA scoring based on EGR1 and its top 30 differentially expressed target genes in CD8^+^ naïve CAR-T cells; middle: ssGSEA scoring based on EGR1 and its top 30 differentially expressed target genes in CD8^+^ memory CAR-T cells; right: ssGSEA scoring based on EGR1 and its top 30 differentially expressed target genes in progenitor CD8_Ex CAR-T cells.) (**D**) OS for the low and high ssGSEA score subgroups of AML patients from the TCGA-AML dataset (left: ssGSEA scoring based on BATF and its top 30 differentially expressed target genes in CD8^+^ naïve CAR-T cells; middle: ssGSEA scoring based on BATF and its top 30 differentially expressed target genes in CD8^+^ memory CAR-T cells; right: ssGSEA scoring based on BATF and its top 30 differentially expressed target genes in progenitor CD8_Ex CAR-T cells.) (**E**) Univariate cox regression analysis of AML patients from the TCGA-AML dataset (**F**) Multivariate cox regression analysis of AML patients from the TCGA-AML dataset

JQ1 exerts widespread impact on different subpopulations including CD8_Naïve, CD8_Memory, and progenitor CD8_Ex CAR-T cells. To further elucidate the key TFs driving these changes, we screened for TFs that exhibited consistent alterations across all the three CAR-T cell subsets in response to JQ1 treatment. The results highlighted that in all three subgroups i.e., CD8_Naïve, CD8_Memory, and progenitor CD8_Ex CAR-T cells, under JQ1-treatment, *BATF* was consistently downregulated, while the *EGR1* was consistently upregulated (Fig. 4A). We further found that *EGR1* regulon target genes were upregulated and BATF regulon target genes were downregulated across all three subgroups (Fig. 4B and Fig. S3A, B). These findings suggested that modulation of two critical TFs may serve as a potential mechanism underlying the JQ1-mediated effects in reducing CAR-T cell exhaustion.

We next sought to investigate whether the expression of *EGR1*, *BATF*, and their target genes might impact the prognosis of AML patients. Therefore, *EGR1* and its top 30 target genes, which were differentially expressed in CD8_Naïve CAR-T cells between the JQ1 and control groups, were subjected to ssGSEA scoring (ssGSEA CD8_Naïve_EGR1 score) based on the AML patient datasets in TCGA database. AML patients with higher ssGSEA CD8_Naïve_EGR1 scores exhibited longer OS and better prognosis compared to patients with lower scores (Fig. 4C). Next, ssGSEA scores for the AML patient dataset were determined based on *EGR1* and its top 30 upregulated expression target genes in JQ1-treated CD8_Memory and progenitor CD8_Ex CAR-T cells (ssGSEA CD8_Memory_EGR1 score and ssGSEA progenitor CD8_Ex_EGR1 score). Survival analysis demonstrated that AML patients with a higher ssGSEA CD8_Memory_EGR1 score and ssGSEA progenitor CD8_Ex_EGR1 score had better prognosis (Fig. 4C). Similarly, ssGSEA CD8_Naïve_BATF scores, ssGSEA CD8_Memory_BATF scores, and ssGSEA progenitor CD8_Ex_BATF scores were determined according to *BATF* and its top 30 downregulated target genes in JQ1-treated CD8_Naïve, CD8_Memory, and progenitor CD8_Ex CAR-T cells. Those with lower CD8_Naïve_BATF, CD8_Memory_BATF, and progenitor CD8_Ex_BATF scores had longer OS and better prognosis (Fig. 4D). Univariate Cox regression analysis also confirmed that the scores based on *EGR1* and *BATF*, as well as their target genes, were independent prognostic factors for AML patient outcome (Fig. 4E). Multivariate Cox regression analysis revealed that AML patients with lower progenitor CD8_Ex_BATF scores had better prognosis (Fig. 4F). Finally, by combining the ssGSEA scores for *EGR1*, *BATF*, and their target genes, we found that AML patients who had higher ssGSEA scores for EGR1 and its target genes, but lower ssGSEA scores for BATF and its target genes exhibited the most favorable prognosis (Fig. S3C).

### VJ gene preferences of the TCR repertoire in CAR-T cells treated with JQ1

**Fig. 5:**
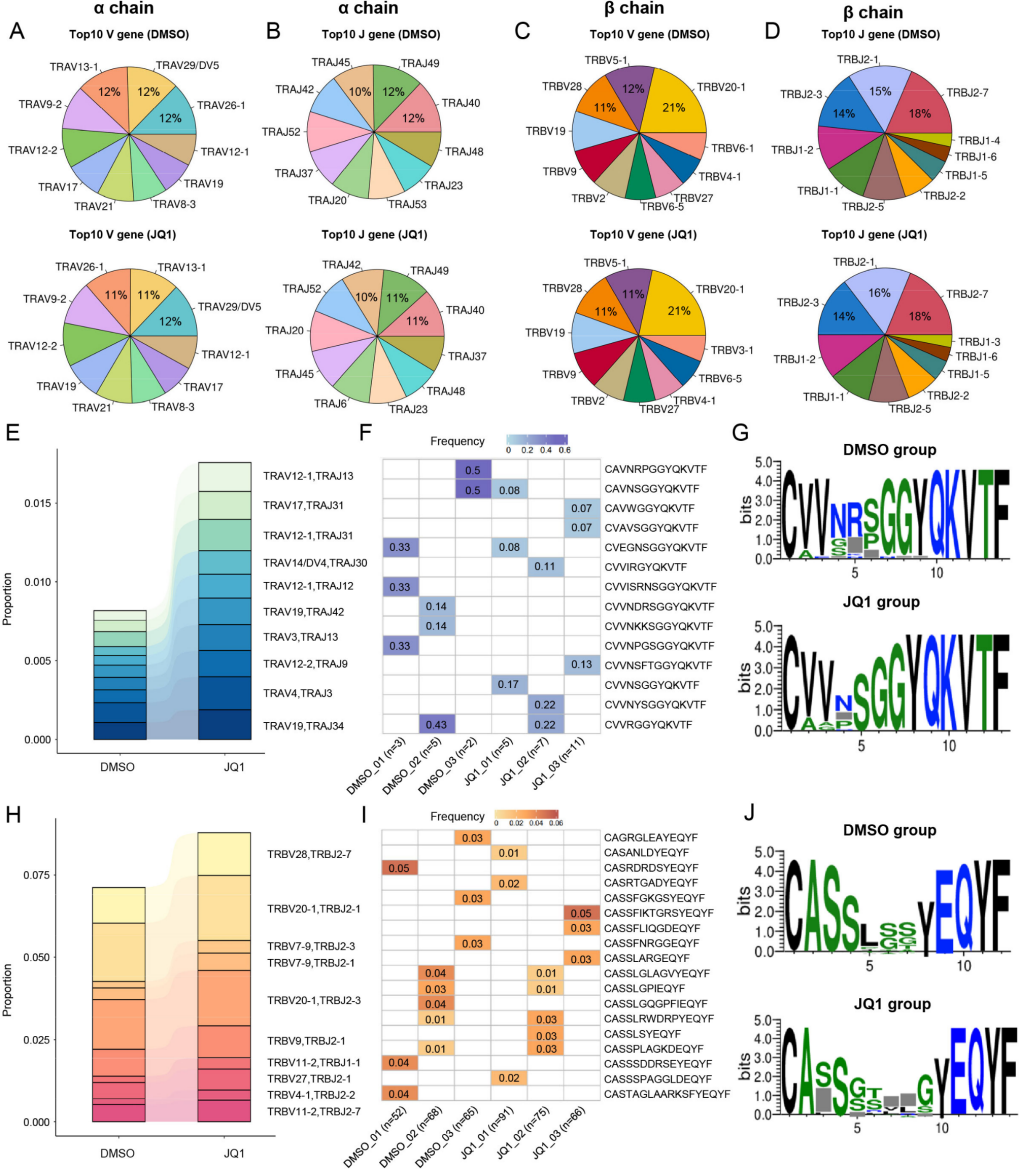
Preferred VJ gene combinations in TCRs from JQ1-treated CAR-T cells (**A**) Pie chart of the top 10 V genes used in the α chain in CAR-T cells in the DMSO group (top) and JQ1 group (bottom) (**B**) The same as plot (A), except including the top 10 J genes used in the α chain (**C**) The same as plot (A), except including the top 10 V genes used in the β chain (**D**) The same as plot (A), except including the top 10 J genes used in the β chain (**E**) Top 10 VJ gene combinations with a higher proportion of α chain combinations in JQ1-treated CAR-T cells (**F**) Heatmap showing the frequency of the top 3 most frequent CDR3 sequences found with the TRAV12-1 and TRAJ13 gene combination in each sample (N represents the total number of unique CDR3 sequences in the TCR α chain found with the TRAV12-1 and TRAJ13 gene combination in each sample.) (**G**) Bias analysis of the CDR3 amino acid motif in the TCR α chain found with the TRAV12-1 and TRAJ13 gene combination (**H**) Top 10 VJ gene combinations with a higher proportion of β chain combinations in JQ1-treated CAR-T cells (**I**) Heatmap showing the frequency of the top 3 most frequent TCR β chain CDR3 sequences found with the TRBV28 and TRBJ2-7 gene combination in each sample (N represents the total number of unique CDR3 sequences in the TCR β chain found with the TRBV28 and TRBJ2-7 gene combination in each sample) (**J**) Bias analysis of the CDR3 amino acid motif in the TCR β chain found with TRBV28 and TRBJ2-7 gene combination

Because TCR repertoire diversity and specific clonal expansion are associated with potential T cell function, we investigated the VJ gene preferences in JQ1-treated CAR-T cells. There were no significant differences between the JQ1-treated group and the control group in terms of number of TCR clonotypes, proportion of unique clonotypes, or TCR clonotype diversity (Fig. S4A-D). The lengths of the CDR3 regions in the α and β chains of the TCRs were also similar between the two CAR-T cell groups (Fig. S4E). However, a closer examination of the TCR gene usage patterns revealed notable differences, although the top 10 most frequently used α chain V genes in the JQ1-treated CAR-T cells did not significantly differ compared to the control group (Fig. 5A). The usage frequency of the *TRAJ6* gene in the α chain of TCRs was increased in JQ1-treated CAR-T cells (Fig. 5B). Additionally, the JQ1-treated CAR-T cells demonstrated a notable increase in the usage frequency of the *TRBV3-1* and *TRBJ1-3* genes in the β chain (Fig. 5C, D). Further analysis of the α chain VJ combinations revealed that the *TRAV12-1* and *TRAJ14* combination had the largest increase in the JQ1 group compared to the control group (Fig. 5E). Interestingly, JQ1-treated CAR-T cells also displayed enhanced CDR3 diversity within the TCR α chain containing the *TRAV12-1* and *TRAJ14* combination, leading to differences in the amino acid motifs in their CDR3 regions (Fig. 5F, G). In contrast to control treatment, JQ1-treated CAR-T cells displayed the most pronounced increase in the frequency of the combination of *TRBV28* and *TRBJ2-7* in the β chain (Fig. 5H). Similarly, TCR clonotypes containing the *TRBV28* and *TRBJ2-7* combination in the JQ1 group exhibited enhanced CDR3 diversity, resulting in distinct amino acid motifs (Fig. 5I, J). These observations suggest that JQ1 may also influence the selection and expansion of specific TCR VJ gene combinations, potentially shaping the functional capabilities of CAR-T cells.

## Discussion

CAR-T cells are T cells that have been enabled to express chimeric receptors targeting specific tumor-associated antigens and recognize and eliminate targeted tumor cells [34, 35]. CAR-T cell therapy has overcome the limitations of traditional chemotherapy and significantly improved the prognosis of patients with hematological malignancies [36, 37]. Particularly in the treatment of B-cell malignancies, CAR-T cell therapy has demonstrated outstanding therapeutic efficacy [38, 39]. However, in AML, the efficacy of CAR-T cell therapy has remained relatively limited [40, 41]. The application of CAR-T cell therapy also faces some key challenges [42]. One of the most critical challenges is CAR-T cell exhaustion [43]. Therefore, mitigating CAR-T cell exhaustion and maintaining its function are crucial for improving the efficacy of CAR-T cell therapy in the treatment of AML.

Recent studies have shed light on promising pharmacological strategies for enhancing the functionality and persistence of CAR-T cells [44]. For instance, it has been reported that phosphoinositide 3-kinase (PI3K) inhibitors can downregulate the expression of exhaustion-associated genes and increase the proportion of CD8^+^ CAR-T cells with stem-like characteristics, ultimately enhancing their ability to eliminate chronic lymphocytic leukemia cells in vivo [45]. Consistent with these findings, our study has also demonstrated that JQ1, a BRD4 inhibitor, can reverse CAR-T cell exhaustion, but the underlying mechanisms have not been elucidated [29]. In this study, scRNA-Seq also revealed that JQ1 treatment can reduce CAR-T cell exhaustion as evidenced by a decrease in the proportion of exhausted CAR-T cells and significant downregulation of immune checkpoint proteins, including *PD-1*, *LAG-3*, and *TIM-3*. A previous study has demonstrated that the tyrosine kinase inhibitor dasatinib can reverse CAR-T cell exhaustion not only by downregulating the expression of exhaustion-associated genes but also by upregulating the expression levels of genes related to naïve or memory T cells [46]. Therefore, we focused on naïve and memory CAR-T cells and found that JQ1 treatment led to a higher proportion of these subpopulations. Importantly, the genes upregulated in naïve and memory CD8^+^ CAR-T cells treated with JQ1 were associated with key T cell functionalities, such as proliferation, activation, and differentiation. These data suggested that JQ1 may enhance the functionalities of naïve and memory CAR-T cells, ultimately improving their therapeutic potential.

Accumulating evidence has indicated that exhausted T cells can be divided into distinct subsets with varying transcriptional and epigenetic features [47]. It is known that the function of exhausted T cells is relatively different in each of its distinct stages [21]. Changes in subset components may have important implications in the prognosis and treatment response of patients with hematological malignancies. Our previous study has demonstrated a skewed distribution for the four discrete stages of exhausted CD8^+^ T cells in different disease statuses and highlighted that a higher proportion of progenitor exhausted CD8^+^ T cells may be associated with a more favorable outcome for B-ALL patients [48]. Importantly, Li et al. demonstrated that the combination of the demethylating agent decitabine and anti-PD-1 antibody can promote the activation and expansion of progenitor exhausted CD8^+^ T cells, effectively suppressing tumor growth in mice [21]. These findings underscore the therapeutic potential of targeting specific exhausted T cell subpopulations. In this study, we found that exhausted CD8^+^ CAR-T cells include a different percentage of progenitor, terminal, and proliferating exhausted cells in the different groups. Notably, the progenitor exhausted CD8^+^ CAR-T cells in the JQ1 group demonstrated a significant increase together with enhanced proliferation, activation, and differentiation capacity. Together, these results highlight the importance of understanding the diverse states of the exhausted T cell populations. Strategies to maintain progenitor exhausted CAR-T cells and block their terminal differentiation may be a promising approach for improving the efficacy of CAR-T cell therapy for hematological malignancies.

The results described above suggest that JQ1 can reprogram naïve, memory, and progenitor exhausted CD8^+^ CAR-T cells, thereby enhancing the functionality of CAR-T cells. Thus, we further sought to elucidate the potential mechanisms underlying this phenotypic and functional remodeling. We found that in naïve, memory, and progenitor exhausted CD8^+^ CAR-T cells treated with JQ1, the activity and expression of *BATF* were downregulated, while the activity and expression of *EGR1* were upregulated. Furthermore, the genes regulated by *BATF* and *EGR1* were associated with the phenotypes of naïve, memory, and exhausted T cells as well as proliferation, activation, and differentiation. Previous studies have demonstrated that *BATF* is an important TF associated with CAR-T cell exhaustion. A study has shown that *BATF* can suppress CAR-T cell function, and depletion of *BATF* can enhance the antitumor capability of CAR-T cells [49]. This result is due to the association of *BATF* with the upregulation of exhaustion-related genes and regulation of genes involved in the development of effector and memory T cells. Similarly, there are also studies reporting that *EGR1* plays an important role in T cell differentiation and activation [50]. *EGR1* has been linked to the enhancement of T cell function through upregulation of key genes, including interleukin-2 (IL-2), tumor necrosis factor (TNF), *CD154*, and interleukin-2 receptor (IL-2r) [51–53]. Therefore, JQ1 may potentially reverse CAR-T cell exhaustion by downregulating *BATF* and upregulating *EGR1*.

The expression levels of genes associated with T cell exhaustion have been found to correlate with the prognosis of patients with hematological malignancies. Our previous research also found that high expression levels of *PD-1*, *PD-L1*, and PD-ligand 2 (PD-L2) were associated with poorer OS in AML patients [54]. Therefore, we further evaluated the significance of *EGR1* and *BATF* and their target genes in the prognostic assessment of AML patients. Interestingly, AML patients exhibiting a higher ssGSEA score for *EGR1* and its target genes, but lower ssGSEA score for *BATF* and its target genes, had longer OS and better prognosis. These scores, based on the expression levels of *EGR1* and *BATF* and their target genes, were independent prognostic factors for AML patient survival. Overall, these findings highlight the potential of targeting *BATF* and *EGR1* as a strategy to improve the efficacy of CAR-T cell therapy. Further investigation of the precise molecular pathways regulated by these transcription factors in the context of CAR-T cell exhaustion may provide valuable insight into the development of more effective CAR-T cell-based immunotherapies.

However, this study has some limitations. We need to further investigate how JQ1 influences the phenotypes and functionalities of CAR-T cell subpopulations through additional experiments. Furthermore, the roles of the key transcription factors *BATF* and *EGR1* also need to be validated.

## Conclusions

The BRD4 inhibitor JQ1 can reduce CAR-T cell exhaustion and maintain naïve, memory, and progenitor exhausted CD8^+^ CAR-T cells, mitigating their terminal differentiation. This BRD4 inhibitor can also enhance the proliferation, activation, and differentiation capacities of the CAR-T cell subpopulations. Lastly, to our best knowledge, we for the first time found that a BRD4 inhibitor can reduce CAR-T cell exhaustion by downregulating *BATF* activity and expression while upregulating *EGR1* activity and expression, providing a novel strategy for enhancing the efficacy of CAR-T cell therapy.

## Electronic supplementary material

Below is the link to the electronic supplementary material.

Supplementary Material 1

Supplementary Material 2

Supplementary Material 3

Supplementary Material 4

Supplementary Material 5

## Data Availability

No datasets were generated or analysed during the current study.

## Abbreviations

AML: Acute myeloid leukemia
BATF: Basic leucine zipper ATF-like transcription factor
BCLAF1: BCL2 associated transcription factor 1
BRD: Bromodomain protein
BRDT: Bromodomain testis-specific protein
CAR-T: Chimeric antigen receptor T
CCNB1: Cyclin B1
CCNB2: Cyclin B2
CCR7: Chemokine receptor 7
CTLA4: Cytotoxic T-lymphocyte associated protein 4
CUX1: CUT-like homeobox 1
EGR1: Early growth response 1
GATAD1: GATA zinc finger domain containing 1
GO: Gene Ontology
GSEA: Gene Set Enrichment Analysis
H1FX: H1 histone family member X
HOPX: Homeodomain-only protein homeobox
IKZF1: Ikaros family zinc finger 1
IL-2: Interleukin-2
IL-2r: Interleukin-2 receptor
IRF7: Interferon regulatory factor
KEGG: Kyoto Encyclopedia of Genes and Genomes
LAG-3: lymphocyte activation gene-3
MAF: Musculoaponeurotic fibrosarcoma
MKI67: Proliferation marker protein Ki-67
OS: Overall survival
PD-1: Programmed cell death 1
PD-L1: PD- ligand 1
PD-L2: PD- ligand 2
PI3K: Phosphoinositide 3-kinase
PKM: Pyruvate Kinase M
POU2F2: POU class homeobox 2
RXRA: Retinoid X receptor alpha
scRNA-Seq: Single-cell RNA sequencing
scTCR-Seq: Single-cell T cell receptor sequencing
STAT1: Signal transducer and activator of transcription 1
TCF7: T-cell factor 7
TFs: Transcription factors
TCGA: The Cancer Genome Atlas
TIM-3: T cell immunoglobulin mucin-domain-containing-3
TCR: T cell receptor
TOP2A: Topoisomerase II Alpha
TOX2: Thymocyte selection-associated high mobility group box 2
ZBTB1: Zinc finger and BTB domain containing 1
ZBTB38: Zinc-finger and BTB domain containing 38
ZNF683: Zinc finger protein 683
