# Supplementary figures and images for: BRD4 inhibitor reduces exhaustion and blocks terminal differentiation in CAR-T cells by modulating BATF and EGR1
Source: Biomark Res. 2024 Oct 15;12:124. doi: 10.1186/s40364-024-00667-w (PMC11476310; doi:10.1186/s40364-024-00667-w)

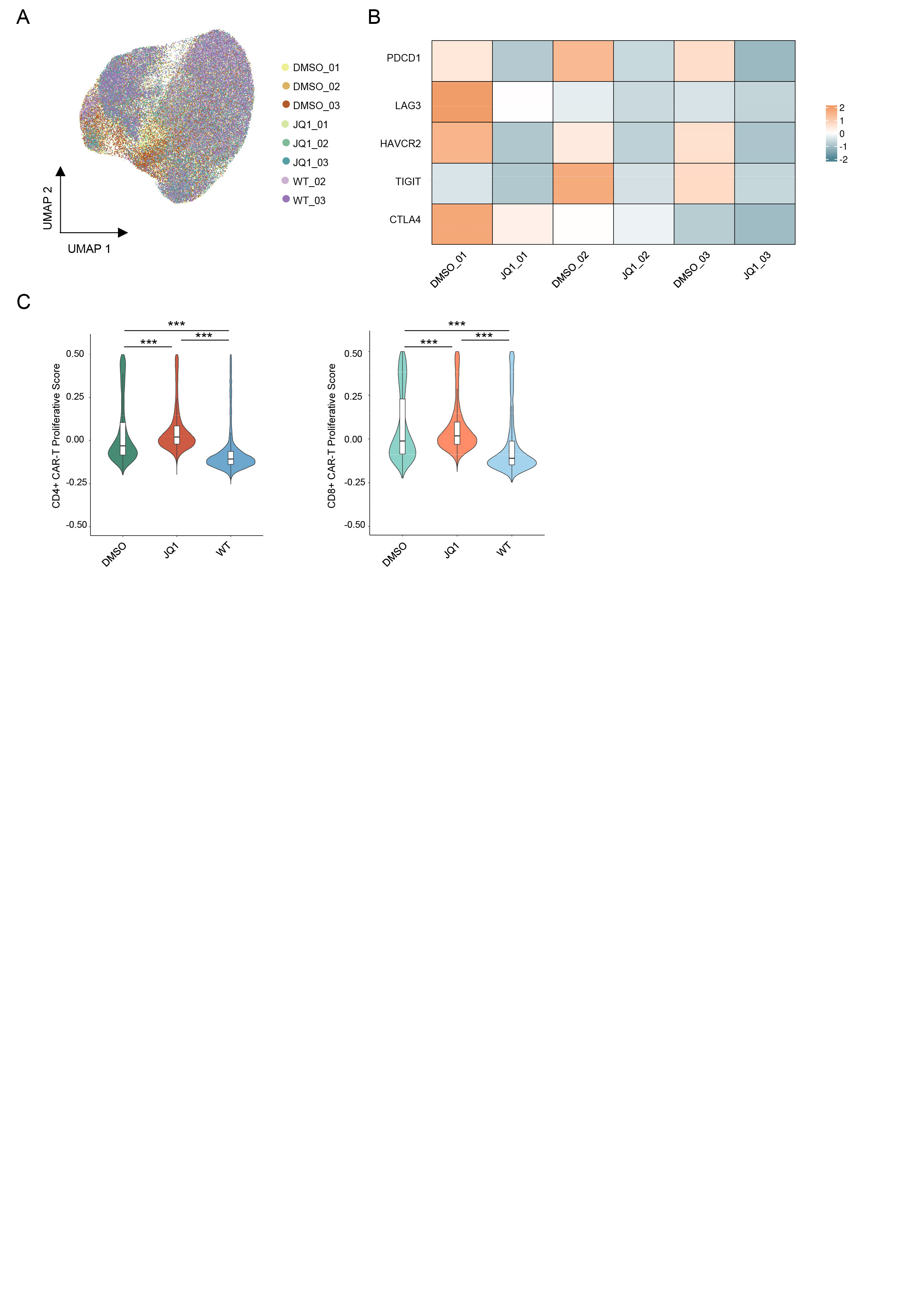

Supplement: Supplementary file 1 — Supplementary Material 1 [file 40364_2024_667_MOESM1_ESM.jpg]

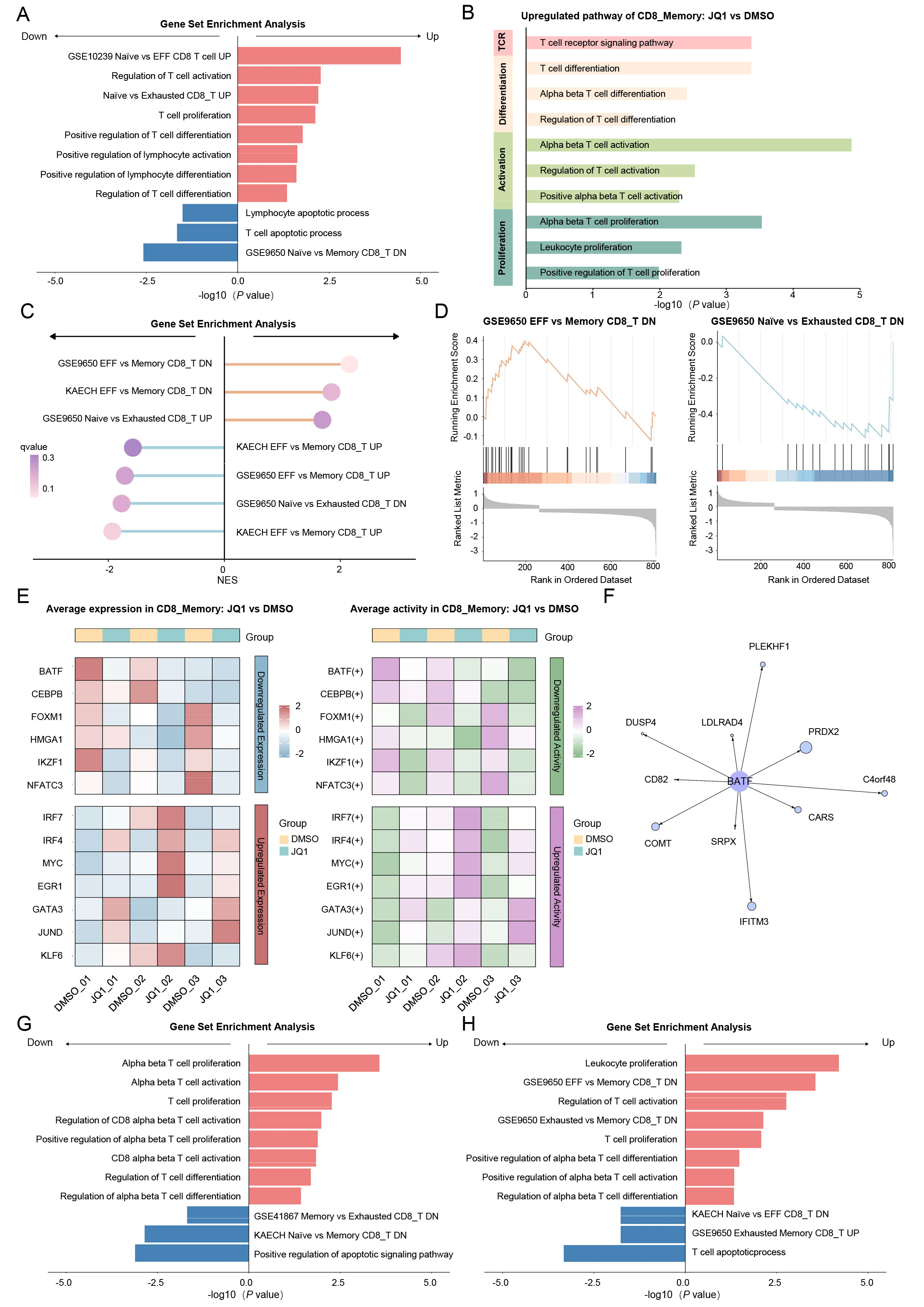

Supplement: Supplementary file 2 — Supplementary Material 2 [file 40364_2024_667_MOESM2_ESM.jpg]

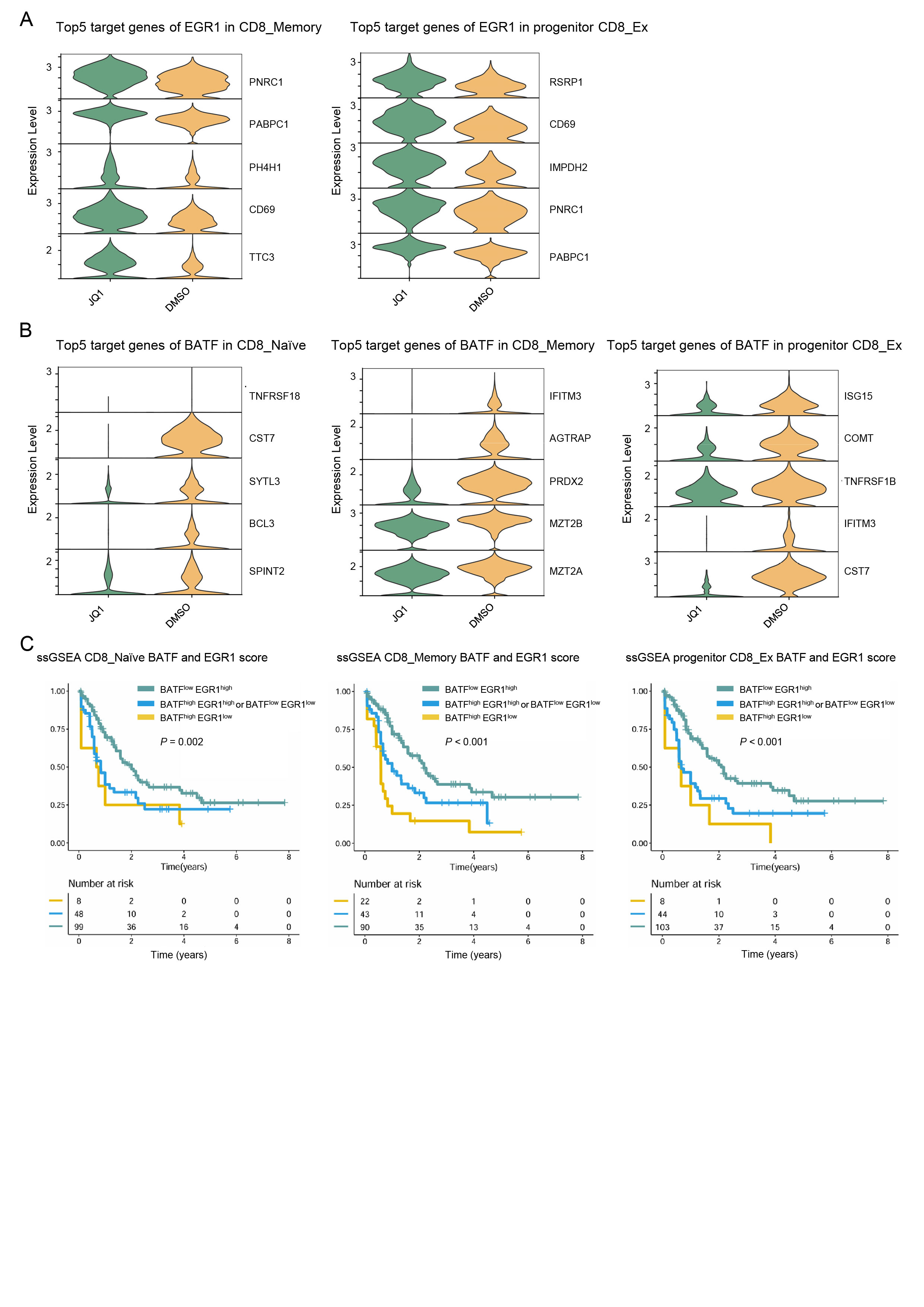

Supplement: Supplementary file 3 — Supplementary Material 3 [file 40364_2024_667_MOESM3_ESM.jpg]

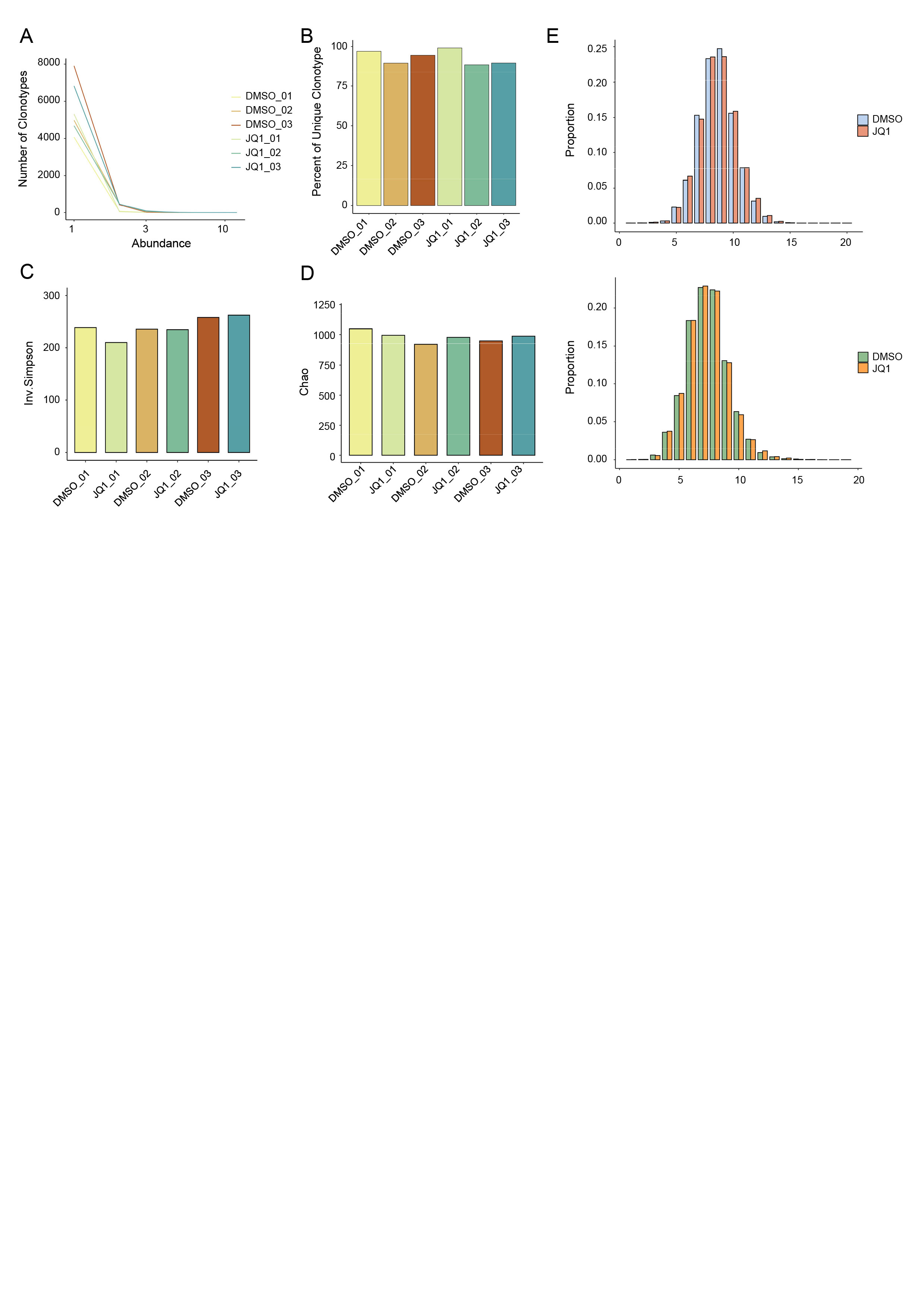

Supplement: Supplementary file 4 — Supplementary Material 4 [file 40364_2024_667_MOESM4_ESM.jpg]
