# Supplementary material for: BRD4 inhibitor reduces exhaustion and blocks terminal differentiation in CAR-T cells by modulating BATF and EGR1
Source: Biomark Res. 2024 Oct 15;12:124. doi: 10.1186/s40364-024-00667-w (PMC11476310; doi:10.1186/s40364-024-00667-w)
Supplement: Supplementary file 5 — Supplementary Material 5 [file 40364_2024_667_MOESM5_ESM.doc]

**Supplementary figure legends**

**Figure S1. Comparison of exhaustion and the proliferation capacity of CAR-T cells between the JQ1 and DMSO group.**

(A) UMAP plot of CAR-T cells scRNA-seq datasets color-coded by sample origin.

(B) Heatmap depicting the expression levels of marker genes related to exhaustion across different samples.

(C) Violin plots of the proliferation scores for CD4^+^ (left) and CD8^+^ (right) CAR-T cells.

**Figure S2. JQ1 reshapes exhausted CAR-T cells.**

(A) Functional enrichment of target genes in the EGR1 and BATF regulons in CD8^+^ naïve CAR-T cells.

(B) KEGG and GO enrichment plot for genes upregulated in CD8^+^ memory CAR-T cells in the JQ1 compared to DMSO group.

(C) GSEA pathways of genes upregulated in CD8^+^ memory CAR-T cells in the JQ1 compared to DMSO group.

(D) GSEA plot showing the expression enrichment of genes related to the GSE9650 EFF vs. Memory CD8_T DN (left) and GSE9650 Naïve vs. Exhausted CD8_T DN (right) pathway in the CD8+ memory CAR-T cells in the JQ1 group.

(E) Heatmap displaying the expression levels (left) and activities (right) of TFs that are consistently upregulated or downregulated in both expression and activity in CD8^+^ memory CAR-T cells in the JQ1 compared to DMSO group.

(F) Network diagram of the BATF regulon (BATF and its top 10 target genes) in CD8^+^ memory CAR-T cells.

(G) Functional enrichment of target genes from the IRF7 and BATF regulons in CD8^+^ memory CAR-T cells.

(H) Functional enrichment of target genes from the MAF and BATF regulons in progenitor CD8_Ex CAR-T cells.

**Figure S3. JQ1 may prolong the OS of AML patients by downregulating BATF and upregulating EGR1.**

(A) Violin plots showing the expression levels of the top 5 differentially expressed target genes in the EGR1 regulon in CD8^+^ memory CAR-T cells (left) and progenitor CD8_Ex CAR-T cells (right).

(B) Violin plots showing the expression levels of the top 5 differentially expressed target genes in the BATF regulon in CD8^+^ naïve CAR-T cells (left), CD8^+^ memory CAR-T cells (middle), and progenitor CD8_Ex CAR-T cells (right).

(C) OS analysis of BATF and EGR1 ssGSEA scoring in AML patients from the TCGA-AML dataset. (left: ssGSEA scoring based on BATF/EGR1 and their top 30 differentially expressed target genes in CD8^+^ naïve CAR-T cells; middle: ssGSEA scoring based on BATF/EGR1 and their top 30 differentially expressed target genes in CD8^+^ memory CAR-T cells; right: ssGSEA scoring based on BATF/EGR1 and their top 30 differentially expressed target genes in progenitor CD8_Ex CAR-T cells.)

**Figure S4. Differences in the TCR repertoire on CAR-T cells between the DMSO and JQ1 group.**

(A) Number of TCR clonotypes for each sample.

(B) Percent of unique TCR clonotypes for each sample.

(C) Bar plot showing Inv.Simpson diversity index scores for the TCR repertoire of each sample.

(D) Bar plot showing Chao diversity index scores for the TCR repertoire of each sample.

(E) CDR3 amino acid sequence length distribution of the TCR repertoire for CAR-T cells in the DMSO and JQ1 group.
